# Why adult patients on antiretroviral therapy miss clinical appointments in rural villages of Limpopo Province, South Africa: An exploratory study

**DOI:** 10.4102/hsag.v27i0.1989

**Published:** 2022-11-04

**Authors:** Mygirl P. Lowane, Rachel T. Lebese

**Affiliations:** 1 Department of Public Health, Faculty of Health Sciences, Sefako Makgatho Health Sciences University, Ga-Rankuwa, South Africa; 2 Department of Nursing, Faculty of Health Care Sciences, University of Venda, Thohoyandou, South Africa

**Keywords:** antiretroviral therapy, missing appointments, non-adherence, patients on ART, scheduled appointment, social support system

## Abstract

**Background:**

Missing clinical appointments while on antiretroviral therapy (ART) is associated with poor adherence to treatment and an increased risk of poor immunological outcomes.

**Aim:**

This study explored the reasons why adults missed clinical ART appointments.

**Setting:**

The study was conducted in community healthcare centres (CHCs) with high rates of missed appointments by ART patients.

**Methods:**

An explorative qualitative research design was used for this study. The population comprised all adult patients on ART who had missed their clinic appointments. The simple random sampling method was used to select sample healthcare centres with high absenteeism from the district health information system. Furthermore, a purposive systematic sampling technique was used to sample ART patients who had missed more than two appointments in a year. Thirty-seven patients were interviewed, as determined by data redundancy, using in-depth individual unstructured interviews, as guided by the following central question: *‘How is it for you to miss your clinic appointments?’* Tesch’s eight steps were used to analyse the data.

**Results:**

Participants cited a lack of family support, financial constraints, nondisclosure of HIV status at the workplace and a lack of patient involvement in scheduling appointments as reasons for nonadherence to ART appointments.

**Conclusion:**

Missed appointments disrupt the functionality of healthcare systems and negatively impact the quality of patient’s care. Patients missing appointment are likely to interrupt HIV care and run a risk of clinical and immunological failure.

**Contribution:**

This study contributes to knowledge as to why patients on ART miss appointments. It will also provide practical guidance to come up with a strategy that will reduce missing appointments and to improve adherence to treatment and health outcomes of patients on ART.

## Introduction

Missed appointments of individuals living with HIV on antiretroviral therapy (ART) programs at healthcare services have become a major concern worldwide (Tweya et al. 2018). The literature describes missing an appointment from the clinic as a failure to attend clinic appointments for more than 5 or 7 days after the date of their next planned visit (Fox et al. 2012; Furtado et al. 2021). It is a burden on essential healthcare systems and has an effect on receiving sustained ART (Cornell et al. 2017; Fox et al. 2018; Larsen et al. 2019; Moosa et al. 2019). Missed appointments are one of the indicators used to measure engagement and retention in HIV care (Pascoe et al. 2018; Pence et al. 2019). Missed appointments result in poor adherence to treatment plans (Dantas et al. 2018). They have a significant impact on the functioning of healthcare institutions because of the increased number of unbooked patients and the influx of unstable people living with human immunodeficiency virus (PLHIV) on treatment (Dantas et al. 2018; Maphumulo & Bhengu 2019). The influx of patients affects the patient–nurse ratio (Maphumulo & Bhengu 2019). Missed appointments negatively affect the quality of HIV care services provided to people living with HIV by the healthcare providers (Mhina et al. 2021).

Missed appointments by HIV patients are associated with delayed viral load suppression, increased HIV burden, an increase in HIV-related opportunistic illnesses and death (Pence et al. 2019). If a patient’s viral loads are unsuppressed, the chance of mutations is high, possibly leading to drug resistance (Meintjes et al. 2017; Mhina et al. 2021). Diverse risk factors for missed visits for HIV medical care were reported. Psychosocial characteristics, such as poor social support and experiences of stigma attached to HIV, were among many other factors found (Kang, Bang & Cho 2018). Bisnauth et al. (2021) highlighted that some reasons why people disengage from healthcare include fear, unstable income and social-interpersonal factors, which range from disclosure to treatment, as well as clinical factors, provider’s attitudes and structural issues (e.g. long waiting times for medical attention at clinics).

Although patients receive counselling from healthcare providers, knowledge and attitudes have been identified as a gap resulting in poor appointment adherence (Georgette et al. 2016). Patients who have a good understanding of their HIV condition are more likely to follow the treatment plans than those who are not fully informed. Some literature mentions that individual factors such as negative attitudes towards healthcare and common mental health disorders such as depression are some of the challenges contributing to disengagement from care (Bisnauth et al. 2021). Adding to those factors associated with missed appointments, living far from the healthcare facility and travelling time were also found to be obstacles to accessing HIV services. Other commitments, being under pressure at the workplace and family duties can also disrupt regular access to health care (Bisnauth et al. 2021).

### Problem statement

Various strategies have been implemented to reduce missing appointments, including physical reminders by community healthcare workers during their support visits, enrolling patients in support groups and recording their return dates on their appointment cards. Despite this effort, ART patients continue to miss scheduled appointments. In Limpopo province, studies regarding missed clinic appointments among patients on ART (particularly in rural primary healthcare facilities) are rare. Most studies have concentrated on poor adherence to ART medication, lost-to-follow-up (LTFU) and no-shows among tuberculosis patients.

### Study purpose

The purpose of the study was to explore patients’ perceptions on missing scheduled ART appointments. This information can be used to inform strategy development to reduce missed appointments and promote treatment adherence among ART patients so that the AIDS epidemic is eliminated by 2030 (Krishnamoorthy, Rehman & Sakthivel 2021).

## Research methods and design

### The design and setting of the study

An explorative, qualitative, descriptive research approach was used to explore the phenomenon of missing two or more appointments. The study was conducted in rural community healthcare centres (CHCs) with high rates of patients who miss clinic appointments in the five districts of Limpopo province, namely Mopani, Vhembe, Capricorn, Waterberg and Sekhukhune. Of the 26 CHCs across the province offering ART service, 15 CHCs with an average 55% missed appointments rate in a quarter were selected.

### Sampling of participants

The study sample consisted of adult ART patients, aged 18 years or older who were on treatment for more than 12 months and had missed two or more appointments in a year during the period of the study. Participants were selected using a nonprobability purposive sampling approach. A list of patients to participate in the study was generated randomly from the TIER.net system by the data capturer from the 15 selected facilities. The TIER.net system is an electronic HIV patient monitoring database used nationally to register people living with HIV at healthcare facilities (Etoori et al. 2020). The researcher and the patients also agreed upon the date and the location (that is, at the facility or their home) for the interview.

### Data collection

Demographic characteristics were captured from each participant before the actual interview. A digital recorder was used during the interview. Nonverbal cues were observed, and field notes were also taken. Data was collected through individual, unstructured, face-to-face interviews. Each interview lasted approximately 45–75 min. The unstructured interview tool was informed by literature on previous research conducted on missing appointments among HIV patients and the objective of this study (Dantas et al. 2018; Pence et al. 2019; Wachira et al. 2022). The tool was developed in English and translated into Xitsonga, Tshivenda and Sepedi to suit the language diversity of the population in the study.

The main researcher conversant with the three local languages conducted the interviews to enhance participation and understanding. The following central question was used: ‘How is it for you to miss your clinic appointments?’ Probing questions and paraphrasing were carried out by asking follow-up questions where the researcher did not fully understand the response or where the researcher wanted the participant to add details, to ensure expansion and deepening of understanding of the phenomenon. The interviews were concluded after the lead investigator realised that there was no new information and data saturation had been reached.

### Data analysis

The data from the digital recorder were transcribed verbatim and then translated to English by the main researcher. The main researcher requested two professional people (who were not part of the research team) who were conversant in the other two languages (Tshivenda and Sepedi) to verify the translation of Tshivenda and Sepedi into English. Data were analysed using Tesch’s eight steps of open coding (Creswell & Cresswell 2017). The primary researcher scrutinised the transcripts several times to identify the similarities and differences and familiarised herself with the data from the interview. Similar topics were grouped from the list established. These were arranged into main themes and subthemes. The generated list was then abbreviated into codes, and researchers checked whether new categories and codes had emerged. Related topics from the list of categories were reduced and groups were formed. The codes were then arranged alphabetically from the formulated topics and reviewed again to check if there were no new codes that emerged. Preliminary analysis was made by grouping the data belonging together. The data was also looked over by another author before the themes and subthemes were finalised. Coding of all data was done separately by the two authors, and a consensus was reached after a thorough discussion. The main author and co-author then agreed on five themes and four subthemes.

### Data quality

Measurements of trustworthiness were observed to ensure data quality, such as credibility, dependability, conformability, and transferability. This study achieved credibility through prolonged engagement and time spent with the participants during appointments. Data transcribed verbatim from the recordings, field notes and observations were kept for audit and for verification purposes. The researchers collected detailed, in-depth data representing a true reflection of why ART patients missed appointments. The results, conclusions and recommendations of this study were supported by participants’ verbatim quotations. This study compared the findings with other studies reporting the same phenomenon to ensure transferability.

### Ethical considerations

Approval for this study was obtained from the University of Venda Research Ethics Committee and the Limpopo Department of Health Research Ethics. Ethical clearance (reference number SHS/17/PH/13/1608) was granted. Participants were fully informed about the study. Informed consent was obtained before the beginning of the interview, and the signed consent forms were kept separately from the transcripts to ensure anonymity.

## Results

A total of 37 patients on the ART program participated in the study. The majority of participants were women (21). Four participants were aged between 20 and 24 years, and 23 participants were aged between 24 and 44 years. Fewer participants (10) were above 45 years, and men were dominant in this age group. Most of the single participants were men, compared to the 43% of women who were married or had been married at some time. Most of the participants (21) were unemployed, while eight were employed or self-employed. Thirty (30) participants resided more than 2 km away from their respective facilities. Most of the participants did not live alone; they were living with either their parents, relatives or children. The data also showed that a high number of participants (24) who missed clinic appointments had been on ART for more than 2 years.

### Theme 1: Lack of family support

A lack of family support was reported by some of the participants; they reported that their family members did not care about the participants’ HIV-positive status and treatment requirements. The study also found that participants depended on family support and expected feelings of empathy from their family members.

‘My mother knows that I am HIV positive, but she [*mother*] prefers not to talk about it as it has brought shame to the family. She [*mother*] told me that I must find a way to manage this condition on my own.’ (Participant 5, male, 22 years old)

‘I sometimes contact my brothers and sister or sometimes my boyfriends, but I am not often successful. They sometimes switch off their phones so that I can’t reach them.’ (Participant 4, female, 30 years old)

Dysfunctional family households and relationships, as well as a lack of clinic accompaniment, also emerged as subthemes.

#### Subtheme 1: Dysfunctional family households and relations

Some participants reported that they came from dysfunctional families who were not able to provide positive support to adhere to their clinic appointments. The following is what some participants reported as challenges in their families:

‘My parents are not staying together, and my mother does not have money for me to go to the clinic every month. Since I got sick, my father uses that as an excuse for not regularly coming home. He always fights with my mother, stating that she wastes money on a dying person.’ (Participant 9, female, 26 years old)

‘Hmm … if you are sick, people will leave you alone. I was staying with my partner, my mother and my other two siblings. I was working at the time, but now they all moved out back to my grandmother’s home … They didn’t say much, but I can feel that I am becoming their burden to them.’ (Participant 21, male, 20 years old)

Some female participants experienced challenges with their sexual partners, who abandoned them or stopped providing support for clinic attendance.

‘My partner left me while I was pregnant when I told him about my status. I even showed him the pills. I have requested him to go for testing; I heard that he is staying with another woman on the farm where he is working. I have not heard from him in a very long time.’ (Participant 16, female, 28 years old)

‘My partner used to give me money to buy food and clothes for the children. He used to say you must save money for the future and unexpected expenses. Since I told him about my status, I am not getting even a cent from him.’ (Participant 22, female, 44 years old)

#### Subtheme 2: Lack of clinic accompaniment

The data revealed that some participants needed someone to accompany them to the clinic due to various reasons. These included fear of walking long distances alone, a need for compassion or because they were sometimes too sick on the day of the appointment. One participant, a 46-year-old woman, reported that she felt supported when she was accompanied by her partner to her appointments. However, the partner often refused to do so and indicated that he was too busy. A participant said the following:

‘I have no one to accompany me to my appointments. The clinic is too far away. Sometimes I don’t have the money for transport.’ (Participant 31, female, 20 years old)

One participant reported that she could not go to the clinic on her own because she was traumatised by her rape.

‘[*S*]even men gang-raped me while I was walking back home from the clinic, stabbed and left in the bush to die. A herdman rescued me and called other people from the village. Then they called an ambulance, and I was taken to the hospital.’ (Participant 11, female, 21 years old)

She further highlighted that no one in her family agreed to accompany her to the clinic following that incident.

A 57-year-old male participant (37), man indicated that because of his other comorbid conditions (hypertension and diabetes mellitus), he was mostly unwell and became tired before arriving at the clinic. He further indicated that although his son had a vehicle and had been taking him to the clinic, this arrangement ended because he (the father) was diagnosed with HIV. Thus, he often had no one to accompany him to the clinic.

### Theme 2: Insufficient funds resulting in working overtime to generate income

Participants also reported that a lack of transport money interfered with adherence to ART appointments. Thus, they were unable to visit the clinic regularly, as they lived far away from the healthcare facility. When further probed regarding other options for getting money for travelling to the healthcare facility, some participants mentioned that they were relying on their children’s social grants. However, some participants reported alternative ways to reach the clinic. One participant mentioned that:

‘I do not have any other option, except lifts. Sometimes I arrive late at the clinic because of lifts, and the nurses verbally attack me when that happens. So, eh … I become disappointed with the treatment after the effort made to arrive at the clinic.’ (Participant 10, male, 44 years old)

Some participants mentioned that a lack of finances interfered with their ability to comply with their scheduled appointments. They cited socio-economic factors such as poverty, unstable employment and insufficient income as the causes.

‘I don’t have a stable job. I rely on casual part-time jobs, and they are not consistent. Sometimes you find that I am called to do washing and other domestic work on weekdays. So usually I sacrifice the clinic ART appointment dates to generate income for food.’ (Participant 36, female, 42 years old)

Some participants reported that they resorted to working overtime to generate funds to go to the clinic and for other family needs. As a result, they sometimes missed their appointments.

‘Hm! I often miss the appointment dates because I work overtime to boost my income. Sometimes when I am off, I look for casual jobs. That is why I sometimes miss the appointment dates. I only go to the clinic when I have the time, which is once in a while.’ (Participant 1, male, 37 years old)

‘My children are receiving grants but the money is too little. I supplement the social grants by selling oranges and other fruit. As a result, I am often unable to go to the clinic because I must go around to different pay points.’ (Participant 7, female, 33 years old)

### Theme 3: Poor adherence to treatment and appointments to sustain social grant

Even though many financial issues were identified, the issue of qualifying for social grants was the main concern for the participants. Social disability grants were extended to people who were sick with HIV and AIDS-related opportunistic infections that resulted in an inability to look for and take up employment opportunities. These grants aim to assist them with financial support until they become healthy enough to look for jobs or become employed. Some reported that the lack of finances affects them physically, and they became unmotivated to go to the clinic. The data also revealed that participants manipulated the system to continue receiving the disability grants even after they had recovered sufficiently to take up employment. This practice was common among the participants, as shown in the following quote:

‘I don’t go for regular [*ART clinic*] visits because they will notice that I have improved well enough, and they will cut my grant.’ (Participant 17, male, 33 years old)

Four participants revealed the tricks they used to disrupt their treatment plans so that they kept on qualifying for social grants. Two participants mentioned that they would stop going to the clinic for some time, interrupting their medication regimens in order to sustain their social grants. Some mentioned that:

‘I sometimes adhere to treatment after the doctor’s assessment, but towards the end of the year, I stop treatment [*ARVs*] and go for clinic visits, so that when I go for an assessment, they will find that my CD4 count is low.’ (Participant 19, male, 28 years old)

The data further revealed that some participants complained that their disability grants were being terminated because of good adherence to treatment. Therefore, they felt that good adherence to ART disadvantaged them as far as receiving the grant was concerned.

‘My pension was cut because they say I’m no longer sick, so now I must start borrowing money from people and pay it back. I am faced with this challenge because I don’t have money at all.’ (Participant 25, male, 50 years old)

### Theme 4: Nondisclosure of HIV status at the workplace

Eight of the ART patients were employed either as part-time employees, on a contract basis or as domestic helpers. However, the data revealed that only one participant revealed her HIV-positive status to her manager. Several reasons were cited for nondisclosure: participants reported that they were afraid of losing their jobs; some were afraid that the employer would reduce their wages; some feared discrimination and stigma from the employer. Two subthemes emerged, namely fear of losing their jobs as well as stigma and discrimination.

#### Subtheme 1: Fear of losing a job

Five participants mentioned that they were afraid of losing their jobs, as they were constantly reminded not to return to work if they knew that they had underlying chronic conditions. Some decided not to disclose their HIV-positive status because they were afraid that their contracts would not be renewed. Three participants mentioned not having proper migrant documentation, so it would be difficult for them to be employed somewhere else, whereas some participants were frightened by employer comments that showed no accommodation of ill employees.

‘The manager told us the company cannot afford sick leave and staff shortages because it affects productivity and profit.’ (Participant 13, male, 35 years old)

#### Subtheme 2: Fear of stigma and discrimination in the workplace

The interviews explored the issues of stigma and discrimination from the working participants. The fear of stigma and discrimination in the workplace was cited by most participants as the reason for not going to the clinic.

‘When I asked for time off to go to the clinic, my supervisor responded by saying “I hope you are not collecting AIDS pills”. Sometimes he makes comments like “Are you also in the sinking ship of AIDS?”’ (Participant 28, female, 38 years old)

The participant indicated that because of such comments, she was reluctant to ask for time off. One participant shared the treatment he experienced after revealing his HIV-positive status to his employer:

‘I was removed from my position as a shift supervisor but transferred to another position of counting stock in the dispatch room. I wasn’t allowed to do any administrative job, like I previously did. They made it seem like the supervisor’s job is not for sick people.’ (Participant 6, male, 56 years old)

One participant mentioned that he felt discriminated against because of the negative comments from his manager regarding people living with HIV in the workplace. These were comments such as ‘We don’t accommodate sick employees in this institution’, or sometimes the manager would say, ‘There is no room for people living with HIV [or] AIDS here’ (Participant 30, male, 39 years old).

### Theme 5: Lack of involvement in scheduling appointment

The current study also found that there was poor interaction between the patients and nurses. All participants reported that they were not involved in selecting the return date for the next follow-up visit. This theme emerged because most participants mentioned that they did not understand how the appointment was scheduled. Almost all the participants indicated that they were not given any opportunity to participate during the ART initiation session. All of them were simply informed about the return date that was documented on their appointment card, according to the appointment booking system of the facility.

A 45-year-old male participant (3) reported that he was told that he had initiated treatment based on the universal test and treat (UTT) approach, without any explanation of this model. He mentioned that after the nurse completed the clinical records, the nurse took out one box of medication and said to him: ‘These are ARVs, and you are going to take them for as long as you live.’ Then he was given an appointment card on which was written the appointment date, without any explanation. The participant mentioned that ‘[j]udging from the nurse’s attitude, I left without asking any questions’.

Some participants tried to explain that they would not be able to come, as they would be working, but their concern was ignored and they were told that the date scheduled for them on their appointment card was final and it was binding to come. When participants were asked about this matter, some responses were as follows:

‘I noticed that I would be at work on the appointment date given … but when I tried to ask for extra medications so that I could come back after two months, I was told that the policy did not allow them to give anyone extra medication.’ (Participant 24, male, 48 years old)

‘I tried to tell the sister that there was no one to accompany me come to the clinic on the day given, but I did not pursue it further because I could see that the sister was not interested.’ (Participant 14, female, 46 years old)

One participant mentioned that his appointment days were changed without being informed.

‘They [*nurses*] have changed my appointment days. I used to go to the clinic on Mondays, but now I go there on Fridays, sometimes Tuesdays or Thursdays. It is confusing me because I am unable to tell my boss on which day I am supposed to go to the clinic.’ (Participant 18, male, 41 years old)

## Discussion

The study explored the reasons why patients on ART missed clinic appointments. The study found that most participants were concerned about a lack of support from family members. Some felt abandoned and rejected by their family members due to their HIV status. It was noted from some participants’ responses that family members often encountered difficulties providing care and living with a sick person (Da Silva & Tavares 2015). Various studies on family dynamics among HIV-affected families have been conducted in different countries (Goodman et al. 2019; Han et al. 2019; Masquillier et al. 2020; Mukumbang et al. 2019). The literature revealed that supporting people living with HIV is important to improve their quality of life and treatment adherence, and it is associated with their psychological well-being (Thanh, Moland & Fylkesnes 2012). The study is a reminder that in order to promote the quality of support for HIV-positive people, active counselling services that include family members should be provided, and families should be encouraged to participate in the care of their HIV-diagnosed family members. Therefore, healthcare workers must encourage family members to accompany their HIV-positive family members to ART appointments, especially at the beginning of the ART initiation sessions (Xu et al. 2017).

It was also noted that participants who reported rejection were those who disclosed their HIV status to their family members (Evangeli & Wroe 2017; Madiba, Ralebona & Lowane 2021). A study conducted in China about families’ reactions and responses towards HIV among the infected members revealed that most family members were less concerned about the health and feelings of their HIV-positive relatives (Yu et al. 2016). Furthermore, HIV-positive patients also lacked the will to share their suffering or details regarding their infection (Yu et al. 2016). Healthcare providers who work with families affected by HIV must therefore make an effort to reduce the discrimination and stigma associated with HIV-positive families by involving participants in counselling sessions.

Discrimination and stigma may result in poor acceptance of HIV status and self-blame for contracting the disease, thereby leaving patients feeling unmotivated to adhere to their treatment plan and neglecting their health. However, literature has suggested that disclosure is positively associated with adherence self-efficacy, which is related to better adherence (Mi et al. 2020).

Financial constraints emerged as another factor obstructing scheduled visits to the clinic. HIV patients are faced with the challenge of sustaining support for long-term treatment. This study revealed that some of the participants access resources such as food and transport to the health facility by requesting money from family members. In a setting where supportive relations often have competing demands and may be difficult, some patients opted for temporary work and even worked overtime to raise money for meeting their needs and to meet both predictable treatment costs, such as regular transport to the health facility for review and refill, as well as emergency cases, such as managing episodic illnesses, rather than seeking assistance from supportive relations (Nanfuka et al. 2018). Therefore, sustaining support for lifelong treatment requires that patients take deliberate strategies to minimise dependence (Nanfuka et al. 2018).

It was also noted that patients risk their lives in order to be retained in the social grant system. Recent studies regarding the issue of social grants among people living with HIV are limited. This study revealed that some patients still demand that their social grant be reinstated to enable them to buy food and pay for travel costs. In this study, it was revealed that the termination of the grants contributed to ART patients deliberately missing appointments or defaulting on ART to remain on the disability grant system. Similar practices were observed in a study conducted in Tanzania, where some patients deliberately interrupted taking their medication (ARVs) to lower their CD4 count to ensure that they qualified for social grants (Govender et al. 2015). Poverty and social grants among people living with HIV intersected, resulting in frustration, distress and discouragement with adhering to the ART treatment plans (Naidoo, Taylor & Mabaso 2017).

Fear of losing their jobs was one reason mentioned by ART participants for failing to disclose their status. Some patients were afraid of being stigmatised, while others suffered emotional abuse in the form of cruel comments from employers, family members and community members. The literature has consistently revealed that social-related variables, such as stigma and discrimination, disclosure and a lack of social support, have a negative influence on adherence to ART (Azia, Mukumbang & Van Wyk 2016). Other literature has indicated that some people conceal their HIV-positive status because they feared that their coworkers and managers might stigmatise them and breach confidentiality (Moalusi 2018). Nondisclosure of HIV status at the workplace might be due to the fears of persecution and breach of confidentiality (Naidoo et al. 2017). Stutterheim et al. (2017) argue that the workplace is a unique environment where HIV status disclosure occurs, albeit with advantages and disadvantages. For example, the advantage is that it can produce supportive workplace accommodation or reduced physical labour. The downside of it, on the other hand, could be possible workplace discrimination. Policies demonstrating a commitment to confidentiality and nondiscrimination against HIV-positive employees in the workplace environment should be clearly endorsed (Naidoo et al. 2017). An effort to address discrimination in the workplace can be made by mobilising healthcare volunteers to visit the workplaces of individuals with HIV and educate and counsel their coworkers regarding support for PLHIV (Xu et al. 2017).

The challenges of scheduling outpatient appointments have received increased attention (Ahmadi-Javid, Jalali & Klassen 2017). In the present study, it became obvious that restricted engagement between healthcare providers and patients results in communication gaps. Communication gaps occur when providers focus only on the issue related to providing medication but fail to respond empathically to patients’ expressions of emotion (Dang et al. 2017; D’Agostino et al. 2017; Deveugele 2015). This corresponds with the author’s realisation during the interviews that nurses only concentrated on ART initiation rather than engaging ART patients to decide their treatment plan. Fear of healthcare workers’ responses was identified as a concern. As a result, ART patients abstain from participating during the ART sessions. This gap results in patients not understanding the rules of scheduling the appointments. Selecting appointment dates should shift from being provider centred to patient centred. Patients must be encouraged to effectively communicate their needs, concerns and preferences (D’Agostino et al. 2017). In so doing, potential patients at risk of missing appointments will be identified on time, and this will also reduce the LTFU rate among this cohort.

### Limitation of the study

This study is solely restricted to the ART patients missing scheduled appointments. However, it cannot be generalised to other patients who were not involved in this study. The findings of this study also cannot be generalised to other chronic patients who missed appointments, as it can render the study meaningless. The effect of acceptance of HIV positive patients by their family members and patient–provider engagement of ART patients have been neglected in research. Therefore, more studies should be conducted to close the gap of this limitation.

## Conclusion

The participants missed their scheduled appointments due to socio-economic factors, disclosure at the workplace and poor provider–patient relationships that contribute to a poor understanding of ART appointment scheduling. In addition, there is evidence of a negative impact regarding disclosure to family members, where some participants suffered rejection. Missed appointments could disrupt the functionality of healthcare systems and negatively impact the quality of patient care. It reduces access to an adequate healthcare system and disrupts effective disease management for patients who miss their appointments.
